# Obesity is associated with severe disease and mortality in patients with coronavirus disease 2019 (COVID-19): a meta-analysis

**DOI:** 10.1186/s12889-021-11546-6

**Published:** 2021-08-04

**Authors:** Zixin Cai, Yan Yang, Jingjing Zhang

**Affiliations:** National Clinical Research Center for Metabolic Diseases, Metabolic Syndrome Research Center, Key Laboratory of Diabetes Immunology, Ministry of Education, and Department of Metabolism and Endocrinology, The Second Xiangya Hospital of Central South University, Changsha, 410011 Hunan China

**Keywords:** Obesity, COVID-19, Predict, Severity, Mortality

## Abstract

**Background:**

The coronavirus disease 2019 (COVID-19) pandemic has led to global research to predict those who are at greatest risk of developing severe disease and mortality. The aim of this meta-analysis was to determine the associations between obesity and the severity of and mortality due to COVID-19.

**Methods:**

We searched the PubMed, EMBASE, Cochrane Library and Web of Science databases for studies evaluating the associations of obesity with COVID-19.

Odds ratios (ORs) and 95% confidence intervals (CIs) were calculated using random- or fixed-effects models. Meta-regression analyses were conducted to estimate regression coefficients.

**Results:**

Forty-six studies involving 625,153 patients were included. Compared with nonobese patients, obese patients had a significantly increased risk of infection.

(OR 2.73, 95% CI 1.53–4.87; *I*^*2*^ = 96.8%), hospitalization (OR 1.72, 95% CI 1.55–1.92; *I*^*2*^ = 47.4%), clinically severe disease (OR 3.81, 95% CI 1.97–7.35; *I*^*2*^ = 57.4%), mechanical ventilation (OR 1.66, 95% CI 1.42–1.94; *I*^*2*^ = 41.3%), intensive care unit (ICU) admission (OR 2.25, 95% CI 1.55–3.27; *I*^*2*^ = 71.5%), and mortality (OR 1.61, 95% CI 1.29–2.01; *I*^*2*^ = 83.1%).

**Conclusion:**

Patients with obesity may have a greater risk of infection, hospitalization, clinically severe disease, mechanical ventilation, ICU admission, and mortality due to COVID-19. Therefore, it is important to increase awareness of these associations with obesity in COVID-19 patients.

**Supplementary Information:**

The online version contains supplementary material available at 10.1186/s12889-021-11546-6.

## Background

On December 31, 2019, the World Health Organization (WHO) was made aware of an outbreak involving several cases of atypical pneumonia. These cases were subsequently identified as being caused by a novel virus belonging to the coronavirus (CoV) family, called severe acute respiratory syndrome coronavirus 2 (SARS-CoV-2) [1]. On January 30, 2020, the WHO declared an international public health emergency due to infections caused by SARS-CoV-2. On February 20, 2020, the WHO officially named the disease caused by SARS-CoV-2 coronavirus disease 2019 (COVID-19) [2, 3]. COVID-19 has posed a global health threat, causing an ongoing pandemic in many countries and territories, with approximately 6,287,771 confirmed COVID-19 cases and 379,941 deaths [4] as of June 3, 2020. The number of COVID-19 cases has been rising worldwide, and there is increasing global concern about this outbreak [5].

WHO global estimates indicate that 39% of adults are overweight and 13% are obese [6]. Obesity is an increasing worldwide health concern and is regarded as a critical risk factor for various infections, postinfection complications and mortality from severe infections [7]. Obesity has been shown to have deleterious effects on host immunity, which is the primary cause of an increased risk of infection, especially severe infection [7, 8]. Obesity has also been shown to affect lung function in multiple ways that are related to mechanical and inflammatory factors, making obese individuals more likely to suffer from respiratory symptoms and progress to respiratory failure [9].

Accumulating evidence suggests that the group of patients who develop severe COVID-19 may have a higher proportion of obesity than the group with non-severe COVID-19; in some reports, the difference was significant [10–13]. However, a lack of information regarding the global prevalence of obesity in individuals with COVID-19 remains. Investigating the influence of obesity on COVID-19 is of scientific interest. This investigation aimed to review the relationship between obesity and COVID-19. In doing so, we aim to enhance public awareness of the association between obesity and COVID-19. Furthermore, highlighting the possible associations between obesity and COVID-19 could guide those working to control the COVID-19 pandemic.

## Methods

### Literature search

The Preferred Reporting Items for Systematic Reviews and Meta-Analyses of Individual Participant Data (PRISMA-IPD) statement was followed for the performance and reporting of this meta-analysis [14]. Our meta-analysis focused on the relationships between obesity and the mortality due to and severity of COVID-19.

PubMed, EMBASE, the Cochrane Library and Web of Science were carefully searched from inception to January 2021 for the terms “COVID-19” and “novel coronavirus” combined with the terms “obesity” and “BMI” as index words. Two investigators (ZC and YY) independently reviewed the identified abstracts and selected articles for full review. Disagreements were resolved by a third investigator (JZ). The search strategy is described in a supplementary file (Supplementary File 1).

### Inclusion and exclusion criteria

The inclusion criteria were as follows: (1) patients in the studies had confirmed COVID-19; (2) the body mass index (BMI) values were provided; (3) the comorbidities and severity of disease were provided; and (4) the studies were published in English. The exclusion criteria were as follows: (1) case reports, reviews, letters or nonhuman studies; (2) studies written in a language other than English; and (3) studies with insufficient information. Two investigators (ZC and YY) independently selected studies for inclusion, and disagreements were resolved by a third investigator (JZ).

### Data extraction

Data extraction was independently conducted by two authors (ZC and YY) using a standardized data collection form that included the author, year, country, patients, BMI values, and outcomes (infection, hospitalization, severe disease, mechanical ventilation, intensive care unit (ICU) admission, and mortality). The characteristics of these studies are shown in Table 1.

**Table 1:** Characteristics of available studies on the relationship between obesity and COVID-19

Number	Author	Year	Country	Patients	BMI	Outcomes
**1**	**Natasha N**	**2020**	**USA**	**238**	**30**	**1.7 (1.1–2.8) for mortality**
**2**	**Céline**	**2020**	**France**	**347**	**30**	**3.0 (1.0–8.7) for severity**
**3**	**Nikroo**	**2020**	**USA**	**363**	**NA**	**1.23 (0.77–1.98) for mechanical ventilation;1.26 (0.79–1.98) for ICU; 1.03 (0.51–2.09) for mortality**
**4**	**Edgar**	**2020**	**Mexico**	**140**	**NA**	**2.3265 (1.0133–5.3415) for ICU**
**5**	**Bo**	**2020**	**USA**	**58**	**30**	**1.98 (0.56–7.72) for hospitalisation; 2.04 (0.5–8.4) for mortality**
**6**	**Marie E**	**2020**	**USA**	**531**	**30**	**1.9 (1.1–3.3) for hospitalisation**
**7**	**Geehan**	**2020**	**USA**	**463**	**40**	**2.0 (1.4–3.6) for ICU**
**8**	**Eduardo**	**2020**	**Mexico**	**32,583**	**NA**	**6.92 (5.54–8.65) for infection**
**9**	**Michael**	**2020**	**USA**	**1000**	**30**	**1.2911 (0.9478–1.7587) for ICU**
**10**	**Xiao**	**2020**	**USA**	**NA**	**NA**	**0.94 (0.86, 1.02) for mortality**
**11**	**Mark**	**2020**	**UK**	**387,109**	**30**	**1.97 (1.61, 2.42) for hospitalisation**
**12**	**Philip**	**2020**	**USA**	**50**	**NA**	**14.4 (2.7052–76.6517) for severity**
**13**	**Juan**	**2020**	**Bolivia**	**107**	**NA**	**12.125 (1.690–86.948) for mortality**
**14**	**Stefano**	**2020**	**Italy**	**132**	**30**	**1.526 (1.243–1.874) for ICU**
**15**	**J.M.**	**2020**	**Spain**	**172**	**30**	**4.725 (1.6143–13.8302) for ICU**
**16**	**Omar**	**2020**	**Mexico**	**177,133**	**NA**	**1.5790(1.5358–1.6235) for infection**
**17**	**Nicole**	**2020**	**USA**	**928**	**NA**	**0.99 (0.58–1.71) for mortality**
**18**	**Kaveh**	**2020**	**USA**	**770**	**30**	**1.76 (1.24–2.48) for ICU; 1.72 (1.22–2.44) for mechanical ventilation; 1.15 (0.62–2.14) for mortality**
**19**	**Luca**	**2020**	**Italy**	**92**	**30**	**4.19 (1.36–12.89) for mechanical ventilation; 11.65 (3.88–34.96) for ICUs; 0.27 (0.03–2.05) for mortality**
**20**	**Eboni G**	**2020**	**USA**	**3626**	**30**	**1.43 (1.20–1.71) for hospitalization**
**21**	**Frederick S**	**2020**	**USA**	**105**	**30**	**1.2908 (0.5936–2.8071) for severity**
**22**	**Eyal**	**2020**	**USA**	**3406**	**40**	**1.6 (1.2–2.3) for the older population mortality**
**23**	**Andrea**	**2020**	**Italy**	**233**	**NA**	**3.04 (1.42–6.49) for mortality**
**24**	**Annemarie B1**	**2020**	**UK**	**20,133**	**NA**	**1.33 (1.19 to 1.49) for mortality**
**25**	**Qingxian**	**2020**	**China**	**383**	**28**	**3.4 (1.4–8.26) for severity**
**26**	**Jerry Y**	**2020**	**USA**	**67**	**30**	**0.8000 (0.1784–3.5872) for ICU**
**27**	**Markos**	**2020**	**USA**	**103**	**30**	**6.85 (1.05–44.82) for mechanical ventilation; 2.65 (0.64–10.95) for ICU**
**28**	**Arthur**	**2020**	**France**	**124**	**30**	**3.45 (0.83–14.31) for mechanical ventilation**
**29**	**Simon**	**2020**	**UK**	**3802**	**30**	**1.41 (1.04–1.91) for infection**
**30**	**Kenneth I**	**2020**	**China**	**214**	**25**	**6.32 (1.16–34.54) for severity**
**31**	**Ling**	**2020**	**China**	**323**	**30**	**1.2514 (0.3735–4.1935) for severity**
**32**	**Leonidas**	**2020**	**USA**	**200**	**35**	**3.78 (1.45–9.83) for mortality**
**33**	**Christopher**	**2020**	**USA**	**5279**	**30**	**1.8 (1.47 to 2.2) for hospitalisation**
**34**	**Rui**	**2020**	**China**	**202**	**28**	**9.219 (2.731–31.126) for severity**
**35**	**Feng**	**2020**	**China**	**150**	**25**	**2.91 (1.31–6.47) for severity**
**36**	**Matteo**	**2020**	**Italy**	**482**	**30**	**4.96 (2.53–9.74) for ICU; 12.1 (3.25–45.1) for mortality**
**37**	**Malcolm**	**2020**	**France**	**83**	**30**	**6.7879 (2.5923–17.7739) for infection**
**38**	**Mohamed**	**2020**	**USA**	**504**	**30**	**1.3 (1.0–1.7) for mortality; 2.4 (1.5–4.0) for mechanical ventilation**
**39**	**Yudong Peng**	**2020**	**China**	**244**	**24**	**8.5853 (4.1817–17.6260) for mortality**
**40**	**Ming Deng**	**2020**	**China**	**65**	**28**	**14 (2.0799–94.2358) for severity**
**41**	**Marta Crespo**	**2020**	**Spain**	**16**	**NA**	**5 (0.5842–42.7971) for mortality**
**42**	**Danielle Toussie**	**2020**	**USA**	**338**	**30**	**3.0 (1.6–5.6) for infection**
**43**	**Michelle Elias**	**2020**	**France**	**1216**	**30**	**3.31 (1.90 to 5.77) for infection**
**44**	**Sudham Chand**	**2020**	**USA**	**300**	**30**	**1.35 (0.88,2.06) for mortality**
**45**	**Justin S. Brandt**	**2020**	**USA**	**183**	**30**	**0.875 (0.3466–2.2088) for infection**
**46**	**Astrid Lievre**	**2020**	**France**	**1289**	**30**	**1.1461 (0.8165–1.6088) for mortality**

### Data synthesis and statistical analysis

All analyses and plots were performed and generated using STATA software (version 12.0, STATA Corp, College Station, TX, USA). Forest plots were used to illustrate the association between obesity and COVID-19 in the selected studies. We pooled the data and calculated the odds ratios (ORs) and 95% confidence intervals (CIs) for dichotomous outcomes, including infection, hospitalization, severe disease, mechanical ventilation, ICU admission, and mortality. The results of the included studies were assessed with random- or fixed-effects models. The *I*^*2*^ statistic was used to assess the magnitude of heterogeneity—25, 50, and 75% represented low, moderate, and high degrees of heterogeneity, respectively. The choice of the appropriate model was based on the results; a fixed-effects model (inverse variance) was used to pool the data if *I*^*2*^ was < 50%, and a random-effects model (DerSimonian-Laird) was used if *I*^*2*^ was > 50% [15]. Funnel plots were used to screen for potential publication bias. To determine the robustness of the results, a sensitivity analysis was conducted with sequential elimination of each study from the pool. The threshold of statistical significance was set to 0.05.

## Results

### Selected studies and baseline characteristics

**Fig. 1:**
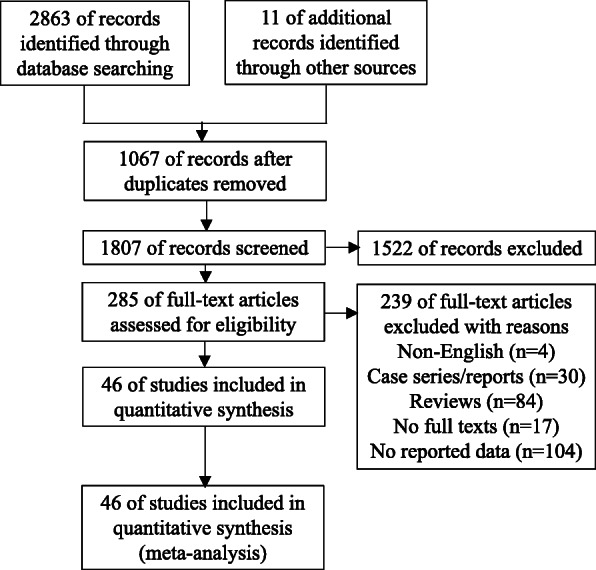
Flow diagram

Overall, 2874 articles of interest were identified in the initial electronic database searches. A total of 1807 duplicate documents were identified. Of these, 285 full-text articles were considered potentially relevant and further assessed for eligibility. After reviewing the titles and abstracts, 239 articles were excluded because they were not written in English, were case series/reports or reviews, did not contain the full text, or had no reported data. The remaining 46 studies were carefully evaluated in detail; these 46 studies met the inclusion criteria and were finally included (Fig. 1). Of the included studies, 18 reported mortality, 10 reported ICU admission, 8 reported the development of severe disease, 7 reported mechanical ventilation, 7 reported infections, and 5 reported hospitalization. Twenty-one studies originated from the USA, 7 originated from China, 5 originated from France, 4 originated from Italy, 3 originated from the UK, 3 originated from Mexico, 2 originated from Spain, and one originated from Bolivia (Table 1). Diagnosis of COVID-19 and definitions of obesity in the included studies were shown in Table 2. Definition of severe COVID-19 used in each study was shown in Table 3. Study design in the included studies were shown in Supplementary Table 1.

**Table 2:** Diagnosis of COVID-19 and definitions of obesity in the included studies

Author	Diagnosis of COVID-19	Definitions of obesity
**Philip Zachariah**	**RT-PCR**	**CDC’s child and teen body mass index**
**Eduardo Hernández-Gardu**	**RT-PCR**	**NA**
**Omar Yaxmehen Bello-Chavolla**	**SARS-CoV-2 testing and signs of breathing difficulty or deaths**	**NA**
**Simon de Lusignan**	**RT-PCR**	**BMI ≥ 30 kg/m2**
**Malcolm Lemyze**	**RT-PCR and typical clinical presentation and imaging features on CT scan**	**BMI > 30 kg/m2**
**Marie E**	**RT-PCR**	**BMI ≥ 30 kg/m2**
**Mark Hamer**	**RT-PCR**	**obese ≥ 30 kg/m2**
**Eboni G**	**RT-PCR**	**BMI ≥ 30 kg/m2**
**Christopher M Petrilli**	**RT-PCR**	**BMI ≥ 30 kg/m2**
**Céline Louapre**	**RT-PCR**	**BMI > 30 kg/m2**
**Frederick S**	**RT-PCR**	**BMI > 30 kg/m2**
**QingxianCai**	**RT-PCR**	**BMI ≥ 28 kg/m2**
**Kenneth I**	**high-throughput sequencing or RT-PCR**	**BMI ≥ 25 kg/m2**
**Ling Hu**	**clinical presentation, characteristic CT image, and/or leukopenia and lymphopenia**	**BMI > 30 kg/m2**
**Rui Huang**	**RT-PCR**	**BMI ≥ 28 kg/m2**
**Nikroo Hashemi**	**RT-PCR**	**NA**
**Stefano Di Bella**	**RT-PCR**	**BMI ≥ 30 kg/m2**
**Kaveh Hajifathalian**	**RT-PCR**	**BMI > 30 kg/m2**
**Luca Busetto**	**RT-PCR**	**BMI ≥ 30 kg/m2**
**Markos Kalligeros**	**NA**	**BMI ≥ 30 kg/m2**
**rthur Simonnet**	**RT-PCR**	**BMI > 30 kg/m2**
**Mohamed Nakeshbandi**	**RT-PCR**	**BMI ≥ 30 kg/m2**
**Edgar**	**RT-PCR**	**NA**
**Geehan Suleyman**	**NA**	**BMI ≥ 40 kg/m2**
**Michael G Argenziano**	**RT-PCR**	**BMI > 30 kg/m2**
**J.M. Urra**	**RT-PCR**	**BMI > 30 kg/m2**
**Matteo Rottoli**	**RT-PCR**	**BMI ≥ 30 kg/m2**
**Jerry Y. Chao**	**RT-PCR**	**BMI ≥ 30 kg/m2**
**Natasha N. Pettit**	**RT-PCR**	**BMI > 30 kg/m2**
**Bo Wang**	**RT-PCR**	**BMI > 30 kg/m2**
**Xiao Wu**	**NA**	**NA**
**Juan Pablo Escalera-Antezana**	**RT-PCR**	**NA**
**Nicole M Kuderer**	**NA**	**NA**
**Eyal Klang**	**RT-PCR**	**BMI > 30 kg/m2**
**Andrea Giacomelli**	**RT-PCR**	**BMI ≥ 30 kg/m2**
**Annemarie B Docherty**	**NA**	**NA**
**Leonidas Palaiodimos**	**NA**	**BMI ≥ 35 kg/m2**
**Yudong Peng**	**RT-PCR**	**BMI ≥ 24 kg/m2**
**Ming Deng**	**RT-PCR**	**NA**
**Marta Crespo**	**NA**	**NA**
**Danielle Toussie**	**RT-PCR**	**BMI > 30 kg/m2**
**Michelle Elias**	**RT-PCR**	**BMI ≥ 30 kg/m2**
**Sudham Chand**	**RT-PCR**	**BMI ≥ 30 kg/m2**
**Justin S. Brandt**	**RT-PCR**	**BMI ≥ 30 kg/m2**
**Astrid Lie `vre**	**RT-PCR**	**BMI ≥ 30 kg/m2**

**Table 3:** Definition of severe COVID-19 used in each study

Author	Year	Definition of a severe form of COVID-19
**Céline Louapre**	**2020**	**7-point ordinal scale (ranging from 1 [not hospitalized with no limitations on activities] to 7 [death]) with a cut off at 3 (hospitalized and not requiring supplemental oxygen)**
**Philip Zachariah**	**2020**	**Severe diseaseas defined by the requirement for mechanical ventilation**
**Frederick S**	**2020**	**defined as admission to the intensive care unit or death**
**QingxianCai**	**2020**	**based on results from chest radiography, clinical examination, and symptoms**
**Kenneth I**	**2020**	**based on the current management guideline**
**Ling Hu**	**2020**	**based initial clinical presentation**
**Rui Huang**	**2020**	**according to guidelines for the diagnosisand treatment of novel coronavirus (2019-nCoV) infection by the national health commission (trial version 5)**
**Ming Deng**	**2020**	**rapid decline in albumin level, the decrease in albumin was accompanied by an increase in D-dimer, which is an indicator of hypercoagulation**

### Viral infection

**Fig. 2:**
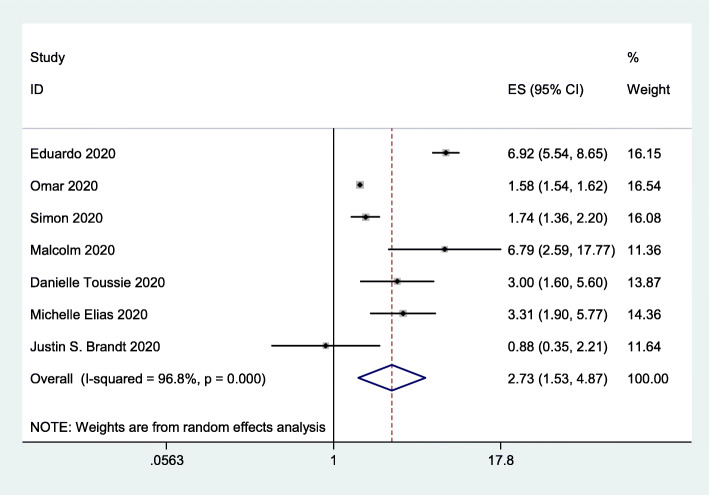
Forest plot comparing the odds of infection with SARS-CoV-2 between obese and nonobese patients

To assess the impact of obesity on viral infection, we included 7 studies [16–22] with 215,338 subjects. The data indicated that obesity significantly increased the risk of viral infection (OR = 2.73, 95% CI 1.53–4.87; *I*^*2*^ = 96.8%; Fig. 2).

### Risk of hospitalization

**Fig. 3:**
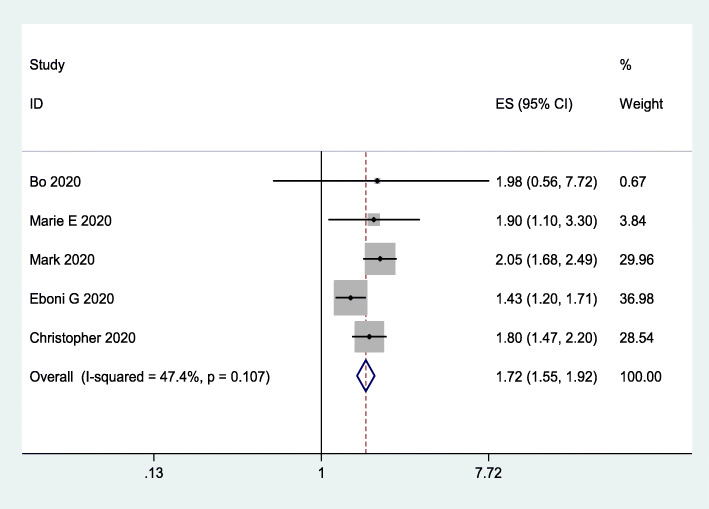
Forest plot comparing the odds of hospitalization for COVID-19 between obese and nonobese patients

To assess the impact of obesity on the risk of hospitalization, we included 5 studies [23–27] involving 396,603 subjects. The data indicated that obesity increased the risk of hospitalization (OR = 1.72, 95% CI 1.55–1.92; *I*^*2*^ = 47.4%; Fig. 3).

### Risk of severe disease

**Fig. 4:**
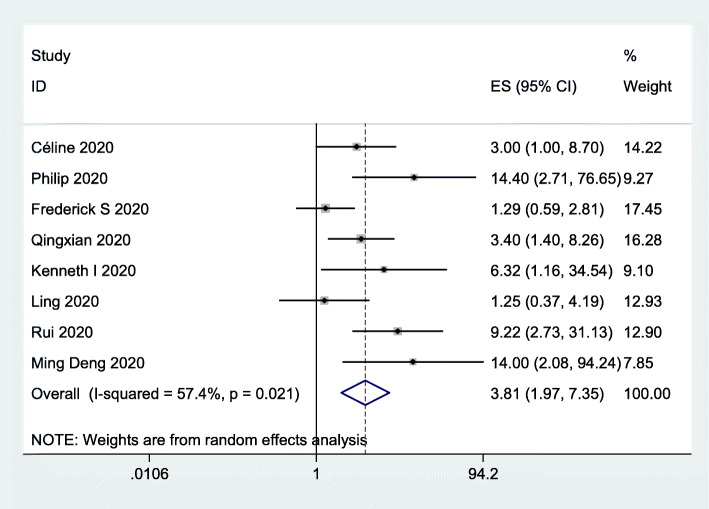
Forest plot comparing the odds of severe COVID-19 between obese and nonobese patients

To assess the impact of obesity on the risk of severe disease, we included 8 studies [10–12, 28–32] involving 1839 subjects. The data indicated that obesity was associated with an increased risk of severe disease (OR = 3.81, 95% CI 1.97–7.35; *I*^*2*^ = 57.4%; Fig. 4).

### Use of mechanical ventilation

**Fig. 5:**
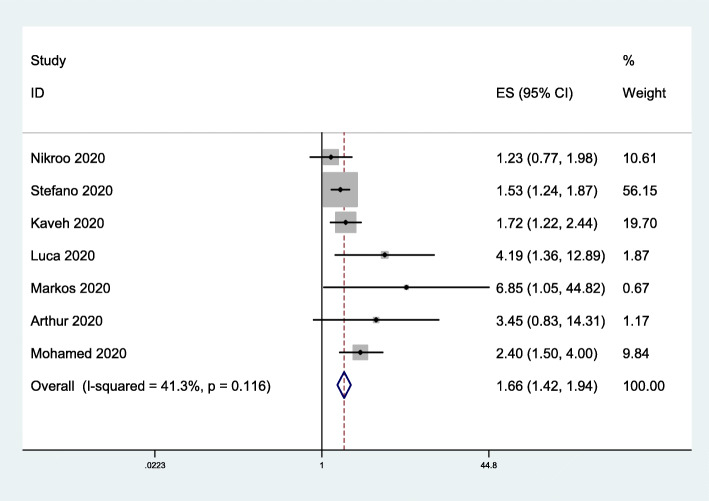
Forest plot comparing the odds of mechanical ventilation due to COVID-19 between obese and nonobese patients

To assess the impact of obesity on mechanical ventilation use, we included 7 studies [33–39] involving 2088 subjects. The data indicated that obesity was associated with the use of mechanical ventilation (OR = 1.66, 95% CI 1.42–1.94; *I*^*2*^ = 41.3%; Fig. 5).

### Risk of ICU admission

**Fig. 6:**
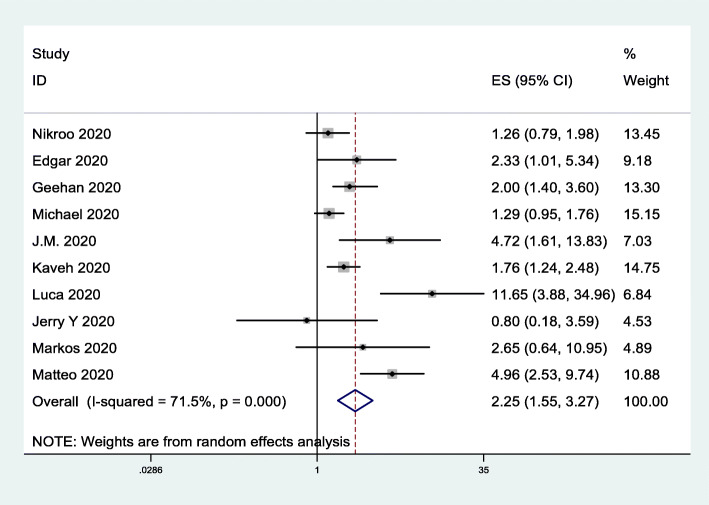
Forest plot comparing the odds of ICU admission due to COVID-19 between obese and nonobese patients

To assess the impact of obesity on the risk of ICU admission, we included 10 studies [33, 35–37, 40–45] involving 3652 subjects. The data indicated that obesity was closely associated with the risk of ICU admission (OR = 2.25, 95% CI 1.55–3.27; *I*^*2*^ = 71.5%; Fig. 6).

### Risk of mortality

**Fig. 7:**
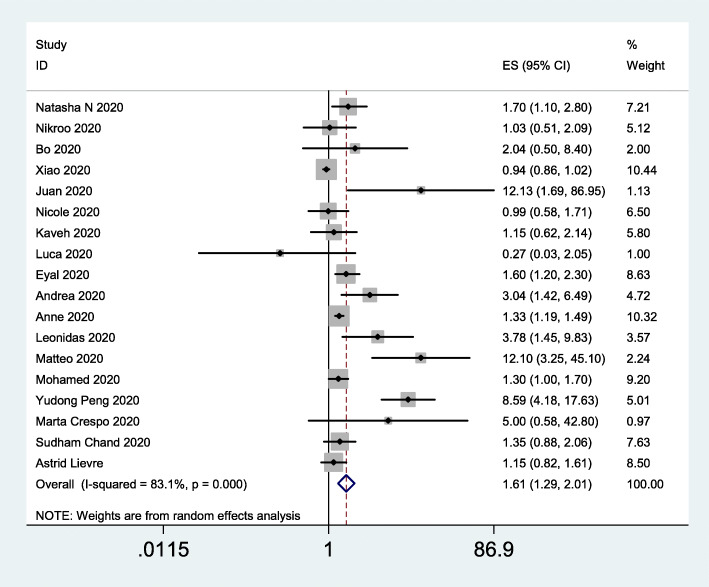
Forest plot comparing the odds of mortality due to COVID-19 between obese and nonobese patients

To assess the impact of obesity on the risk of mortality, we included 18 studies [23, 33, 35, 36, 39, 44, 46–56] [57] involving 29,305 subjects. The data indicated that obesity was significantly associated with the risk of mortality (OR = 1.61, 95% CI 1.29–2.01; *I*^*2*^ = 83.1%; Fig. 7). Univariate meta-regression analysis of possible confounders of COVID-19 outcomes in patients with and without obesity was shown in Table 4.

**Table 4:** Univariate meta-regression analysis of possible confounders of COVID-19 outcomes in patients with and without obesity

lnor	exp (b)	Std. Err.	t	P > |t|	[95% Conf. Interval]
**country |**	**0.58474**	**1.54184**	**−0.2**	**0.844**	**0.0013373,255.6731**
**cons |**	**6.06585**	**31.98394**	**0.34**	**0.741**	**0.0000318,1,157,460**

### Publication bias and sensitivity analysis

**Fig. 8:**
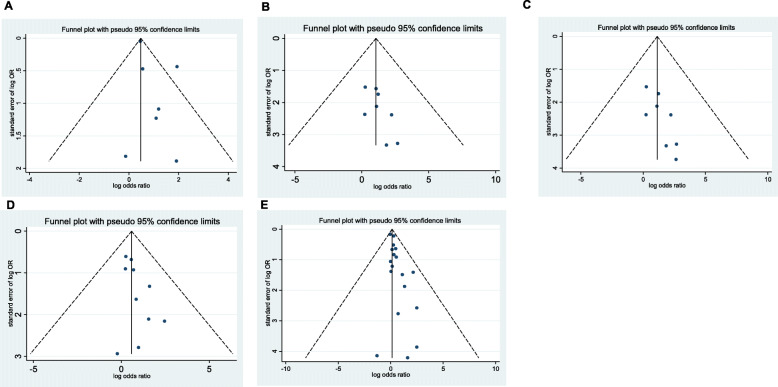
Funnel plot for hospitalization (**A**), severe disease (**B**), mechanical ventilation (**C**), ICU admission (**D**), and mortality (**E**) between obese and non-obese patients

**Fig. 9:**
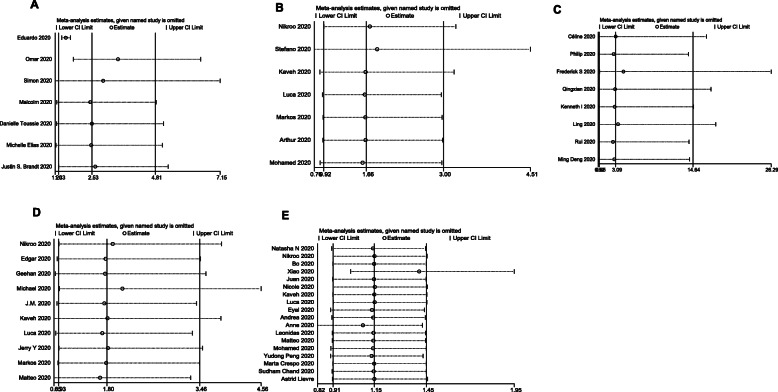
Sensitivity analysis for hospitalization (**A**), severe disease (**B**), mechanical ventilation (**C**), ICU admission (**D**), and mortality (**E**) between obese and nonobese patients

We found no potential publication bias in the studies included in the meta-analysis (Fig. 8). The sensitivity analysis suggested that our results are stable

and reliable (Fig. 9).

## Discussion

We conducted this meta-analysis to determine whether obesity is a predictor of the COVID-19 severity of and mortality. In the present review, we included 46 articles involving 625,153 patients. Obese patients had a significantly increased risk of infection, hospitalization, severe disease mechanical ventilation, ICU admission, and mortality relative to patients of normal weight.

### Mechanisms underlying the association of obesity with the severity of and mortality due to COVID-19

The first mechanism underlying the investigated associations involves adipose tissue (AT). Obesity, usually defined as a BMI > 30 kg/m^2^, is characterized by visceral AT expansion and inflammation [58]. Adipocytes secrete a plenty of factors and hormones that affect many organ systems, including the lungs. Underlying mechanisms of obesity on the severity of COVID-19 may involve abnormalities in the production of adipokines by AT, for example, leptin and adiponectin [59, 60]. Leptin as a cytokine can have pro-inflammatory functions that influences both innate and adaptive immune responses by stimulating the production (interleukin (IL)-2 and tumour necrosis factor-alpha (TNF-α)) and suppressing the secretion of IL-4 and IL-5 [61]. In contrast, adiponectin is adipokine that exerts anti-inflammatory actions that inhibits (TNF-α, IL-6, and nuclear factor-κB) and induces (IL-10 and IL-1 receptor antagonist) [61]. Leptin concentrations are increased, whereas adiponectin levels are decreased in obesity [62, 63]. The imbalance between leptin and adiponectin may result in the development of dysregulated immune response [64].

The second mechanism involves angiotensin-converting enzyme-2 (ACE-2), COVID-19 utilizes the host ACE2 for binding and entry into host cells. The ACE2 expression is highest in AT. The increase of AT in obese patients increases the expression level of ACE2, which may increase their susceptibility to COVID-19 [65].

Third, impaired lung function and higher level of pro-inflammatory Cytokines may collaborate to promote the development of respiratory viral infections in patients with obesity. Obesity reduces thoracic wall compliance, resulting in a reduction in functional residual capacity and favor the development of atelectasis [9, 66].

Finally, obesity results in physiological lung alterations, such as decreased functional residual capacity and hypoxemia [67]. In addition, obstructive sleep apnoea hypopnea syndrome (OSAHS) increases adverse outcomes of COVID-19 [68]. The etiology of OSAHS is complex, and obesity is one of the main causes of the syndrome. OSAHS is related to obesity. About 60–90% of patients with OSAHS are overweight [69], and the incidence rates of OSAHS in the obese patients is near twice that in normal-weight patients [70].

All of the above mechanisms can reasonably explain how obesity increases COVID-19 severity and mortality.

### Implications for strategies to treat patients with obesity

Obesity is a clinical predictor of adverse outcomes in COVID-19 patients; therefore, improved intensive care guidelines for patients with elevated BMI are strongly recommended. Individuals with obesity is an important risk factor for COVID-19, including infection, hospitalization, severe disease, mechanical ventilation, ICU admission, and death. Patients with obesity may require special monitoring. Therefore, obesity patients with COVID-19 require special attention. Additionally, people of obesity should be offered as prioritizing for vaccination of COVID − 19.

Obesity aggravates adverse outcomes in COVID-19 patients, and the occurrence of COVID-19 also leads to an increase in obesity. The public control of the COVID-19 outbreak is mainly about controlling human contact, which affects people’s behavior to a certain extent and contributes to obesity [71]. Isolation susceptibility to incidence of mental illness [72]. Experiencing loneliness can lead to cut down on physical activity [73]. Regular physical activity is important for maintaining body weight. And as economic conditions decline, people turn to cheaper foods, which tend to be higher in calories [74]. While more and more people are cooking at home, food stored is likely to be processed to extend its shelf life. Processed foods are associated with more fat, carbohydrate and calorie intake, which is more likely to lead to weight gain than a healthy diet [75].

Preventing obesity is important. Losing weight usually involves increasing physical activity and limiting caloric intake. It is said that individuals complete ≥300 min/week of physical activity for weight maintenance [76]. People implemented a variety of weight loss strategies, including eating less, increasing physical activity, skipping meals, or taking weight-loss pills or diuretics [77]. Among those trying to lose weight, reducing calorie intake is the most common way [78, 79].

One study found that use of metformin was significantly associated with a reduction in COVID-19 mortality [80]. Several reasons might explain this finding. First, metformin reduces the binding of the SARS-CoV-2 to the receptor [81]. Second, metformin inhibits the mTOR signaling pathway, thus reducing SARS-CoV-2 infectivity and COVID-19 mortality [80]. Third, metformin can the inflammatory response [82]. Additionally, metformin reduces the risk of adverse outcomes in COVID-19 patients by reducing BMI and body weight [83].

Due to the extensive spread of COVID-19, enforced confinement has influenced the lives of individuals in many ways, including working behaviours, psychological factors, sedentary activities, and other harmful effects on life habits [84]. Because of increased stress and boredom, people tend to overeat, resulting in the consumption of additional energy/calories and an increased craving for food [85]. In this regard, COVID-19 has contributed to the occurrence of obesity.

### Theoretical and practical implications

To the best of our knowledge, this is the first meta-analysis to comprehensively assess obesity and COVID-19 outcomes (infection, hospitalization, severe disease, mechanical ventilation, ICU admission, and mortality). Obesity is a risk factor and predictor of serious disease and is a factor in the need for advanced medical care for COVID-19 patients. Basic research is needed to determine the causal relationship between obesity and adverse outcomes of COVID-19. This study has some limitations. First, some indicators, such as the risk of infection, ICU admission, and mortality, had greater degrees of heterogeneity, and subgroup analyses cannot be performed due to the small number of studies on each indicator. However, the trends were consistent across nearly all forest plots. In addition, many of the included articles did not give specific BMI values, and it is not clear how much a specific unit increase in BMI can increase the severity and mortality rate of COVID-19. Third, because this meta-analysis includes data from multiple countries, the criteria for ICU admission and mechanical ventilation usage may not have been uniform. However, the decision to escalate a patient to critical care is primarily based on the judgement of clinicians, as there are no set guidelines at individual sites. Finally, because none of the studies were randomized controlled trials, the causal relationships between obesity and COVID-19 severity and mortality could not be determined.

## Conclusion

Patients with obesity may have a greater risk of COVID-19 infection, hospitalization, clinically severe disease, mechanical ventilation, ICU admission, and mortality. Our results may prompt clinicians to pay particular attention to obese patients when treating COVID-19.

## Supplementary Information

**Additional file 1.** Full electronic search performed in multiple international databases.

**Additional file 2: Table S1.** Study design.

## Data Availability

The datasets used and/or analyzed during the current meta-analysis are available from the corresponding author upon reasonable request.

## Abbreviations

COVID-19: Coronavirus Disease 2019
ORs: Odds ratios
CIs: confidence intervals
WHO: World Health Organization
CoV: coronavirus
SARS-CoV-2: severe acute respiratory syndrome coronavirus 2
PRISMA-IPD: Preferred Reporting Items for Systematic Reviews and Meta-Analyses of Individual Participant Data
BMI: body mass index
ICU: intensive care unit
AT: adipose tissue
IL: interleukin
TNF-α: tumour necrosis factor-alpha
ACE-2: angiotensin-converting enzyme-2
OSAHS: obstructive sleep apnoea hypopnea syndrome
