# Supplementary material for: Obesity is associated with severe disease and mortality in patients with coronavirus disease 2019 (COVID-19): a meta-analysis
Source: BMC Public Health. 2021 Aug 4;21:1505. doi: 10.1186/s12889-021-11546-6 (PMC8334342; doi:10.1186/s12889-021-11546-6)
Supplement: Supplementary file 1 — Additional file 1. Full electronic search performed in multiple international databases. [file 12889_2021_11546_MOESM1_ESM.docx]

# Full electronic search

Full electronic search performed in multiple international databases.

- PubMed
- Embase
- Cochrane Library
- Web of Science

|  |
| --- |
|  |

### PubMed search formula

#1) “Search ((COVID-19) OR novel coronavirus)

#2) “Search ((obesity) OR BMI)

#3) “Search ((COVID-19) OR novel coronavirus) AND ((obesity) OR BMI)

### Web of science search formula

Timespan = All Years(1950-2020), Search language to use = English

#1) TS = “COVID-19” OR TS = “novel coronavirus”

#2) TS = “obesity” OR TS = “BMI”

#3) #2 AND #1

### Embase Search

#1) ‘COVID-19’/exp OR ‘novel coronavirus’

#2) ‘obesity’/exp OR ‘BMI’ /exp

#3) #1 AND #2

#4) #3 AND (‘clinical trial’/de OR ‘randomized controlled trial’/de)

#5) #3 AND (‘clinical trial’/exp OR ‘clinical trial’ OR ‘randomized controlled trial’/exp OR ‘randomized controlled trial’)

#6) #3 AND (‘clinical trial’/exp OR ‘clinical trial’ OR ‘randomized controlled trial’/exp OR ‘randomized controlled trial’) AND ([controlled clinical trial]/lim OR [randomized controlled trial]/lim)

#7) #3 AND (‘clinical trial’/exp OR ‘clinical trial’ OR ‘randomized controlled trial’/exp OR ‘randomized controlled trial’) AND ([controlled clinical trial]/lim OR [randomized controlled trial]/lim) AND [embase]/lim

Cochrane Library Search formula

#1) COVID-19 or novel coronavirus

#2) obeisty or BMI

#3) #1 and #2 and #3

#4) MeSH descriptor: [COVID-19] explode all trees

#5) MeSH descriptor: [novel coronavirus] explode all trees

#6) MeSH descriptor: [obeisty] explode all trees

#7) MeSH descriptor: [BMI] explode all trees

#8) #4 or #5

#9) #6 or #7

#10) #8 and #9

#17) #10 or #3

#18) #17 or #3 in Trials
